# DRL-Based LEO Constellation Design for Regional Navigation Enhancement

**DOI:** 10.3390/s26175607

**Published:** 2026-09-03

**Authors:** Zixuan Rui, Fangling Zeng, Xiaofeng Ouyang, Lichao Chen

**Affiliations:** 1 College of Electronic Countermeasures, National University of Defense Technology, Hefei 230037, China; ruizixuan17@nudt.edu.cn (Z.R.); chenlic@nudt.edu.cn (L.C.); 2 Space Security Research Center, Space Engineering University, Beijing 101416, China

**Keywords:** constellation design, multi-objective optimization, double deep Q-network, reinforcement learning, GDOP

## Abstract

Designing Low Earth Orbit (LEO) constellations for applications like Positioning, Navigation, and Timing (PNT) is a challenging multi-objective optimization challenge. Conventional metaheuristics often suffer from premature convergence due to their reliance on static adaptive rules, limiting their effectiveness in complex search space. To address this limitation, we proposed a hybrid framework where a Double Deep Q-learning Network (DDQN) agent learns a policy to adaptively control the key parameters of a Particle Swarm Optimization (PSO) algorithm. The proposed framework formulates Walker constellation optimization as an sequential parameter control problem. Based on constellation performance feedback, the DDQN controller jointly selects the inertia weight and acceleration coefficients of PSO, guiding the PSO to more effectively balance exploitation and exploration. In a regional design case for China, our algorithm demonstrated superior performance. Compared to a 120 satellites benchmark constellation, the optimized constellation achieved a 27% reduction in Geometric Dilution of Precision (GDOP), a 27.6% enhancement in navigation accuracy, and a 5% increase in coverage multiplicity. This work establishes a robust methodology for the automated and intelligent design of LEO systems, validating the potential of deep reinforcement learning methods for complex aerospace optimization problems.

## 1. Introduction

The growing demand for high precision Positioning, Navigation, and Timing (PNT) and satellite communication services has drawn increasing attention to Low Earth Orbit (LEO) constellations. Ongoing mega constellation initiatives, such as Starlink, GuoWang, and CentiSpace, envision the deployment of thousands to tens of thousands of satellites [1,2]. Compared with traditional Medium Earth Orbit (MEO), LEO satellites provide stronger received signals and faster geometric variation, which can improve positioning robustness and accelerate Precise Point Positioning (PPP) convergence [3,4]. Early studies established the feasibility of LEO enhanced GNSS (LeGNSS) and quantified PPP benefits under different constellation configurations [5]. More importantly, recent in-orbit demonstrations have begun to validate these gains with real measurements. Using observations from two CentiSpace experimental satellites, Li et al. reported measurable improvements in PPP precision and a substantial reduction in convergence time [6,7]. Further studies indicate that LEO enhancement is likely to become an important component in future PNT architectures [8].

Despite this promise, designing a LEO constellation specifically for regional navigation enhancement remains a challenging task. In practice, regional augmentation requires not only favorable average geometry but also robust service continuity under strict operational constraints. This leads to a highly constrained multi-objective optimization problem that must balance competing objectives such as coverage and geometry metrics [9,10,11].

To address these challenges, we incorporate deep reinforcement learning (DRL) into the regional LEO constellation design loop as an adaptive control mechanism. We formulate Walker constellation optimization as an sequential control problem. The DDQN controller uses constellation performance feedback to jointly select the inertia weight and the cognitive and social acceleration coefficients of Particle Swarm Optimization (PSO).

The main contributions of this work are summarized as follows:

- We formulated regional LEO navigation augmentation constellation design as a constrained multi-objective optimization problem with practical service requirements.
- We proposed a DRL-based swarm intelligence framework (PSO-DDQN) that adaptively tunes PSO search behavior, improving optimization efficiency and solution quality under strict constraints.
- We conducted regional experiments over China and benchmarked optimized constellations against a representative reference configuration, demonstrating improved coverage robustness and coverage performance.

The remainder of this paper is organized as follows. Section 2 reviews the related work. Section 3 formulating the constellation optimization problem and presenting the proposed PSO-DDQN framework. Section 4 describes the experimental design, setup, and agent training dynamics. Section 5 reports the specific analysis results, followed by a comprehensive discussion in Section 6. Finally, Section 7 concludes the paper.

## 2. Related Work

### 2.1. Walker Constellation Optimization

Walker constellation design involves the selection of orbital and structural parameters that jointly determine regional coverage, navigation geometry, and deployment cost. Analytical and enumeration methods can be effective when the number of design variables is limited, but their computational cost increases rapidly as additional orbital variables, performance objectives, and operational constraints are introduced. Existing studies have explored a range of optimization approaches to address constellation design beyond purely analytical templates [12]. Evolutionary and swarm-based methods are particularly common due to their flexibility in handling non-convex objectives. For example, strategies based on genetic algorithm have been applied to improve visibility robustness and geometry [13,14,15,16], while multi-objective PSO and differential evolution variants have been used to handle constellation design and constraint satisfaction [17,18,19]. Bayesian optimization has also been investigated for regional enhancement to improve search efficiency [20].

These studies demonstrate the effectiveness of swarm-based optimization for constellation design. Nevertheless, the resulting search performance remains sensitive to the internal control parameters of the optimizer. This issue is especially relevant to Walker constellation design because discrete structural variables and continuous orbital variables jointly affect coverage, navigation geometry, and constellation size. The inappropriate balance between exploration and exploitation can lead to premature convergence or inefficient evaluation of candidate constellations.

### 2.2. Adaptive and RL-Assisted PSO

PSO is attractive for constellation design problem because of its simple update mechanism and relatively low implementation cost. Nevertheless, its performance is sensitive to the inertia weight and the cognitive and social acceleration coefficients. A variety of parameter control strategies have been developed to improve the adaptability of PSO. Early approaches generally used deterministic schedules, adaptive inertia weights, fuzzy rules, or information on the history of success to balance exploration and exploitation. Although these methods reduce the dependence on fixed coefficients, their adjustment rules are usually specified before optimization and may not generalize to different search states or problem structures.

Reinforcement learning provides an alternative approach in which policies are learned through interactions with the optimization process. Liu [21] used Q-learning to select PSO parameters according to the optimization state, while Wu and Wang [22] employed a policy-gradient strategy to improve particle updating. Yin et al. [23] developed a DDPG-based parameter adaptation method that controls the PSO coefficients using iteration progress, stagnation, and swarm diversity. Li et al. [24] used reinforcement learning to select velocity-update and neighborhood mutation strategies. More recently, Aoun [25] combined search-state identification with a deep Q-network for online self-tuning of PSO parameters. Liu et al. [26] further introduced a PPO-based adaptive PSO with multi-source learning and a dynamic reward mechanism. These developments demonstrate the continuing relevance of reinforcement learning for adaptive swarm optimization.

### 2.3. Research Gap and Positioning

These studies demonstrate the potential of learning-based parameter control; however, their states and rewards are usually derived from generic fitness values and do not explicitly represent the characteristics of constellation design. The constellation design studies above generally employ fixed PSO parameters or predefined adaptation schedules. Consequently, they do not learn a feedback policy that maps the current constellation performance to an appropriate balance between exploration and exploitation. Therefore, the remaining research question is how to construct a specific constellation state and action feedback mechanism and integrate it with constellation evaluation. DRL enables an agent to learn decision policies through interaction with the optimization environment [27,28]. Building on this idea, we introduce a hybrid PSO-DDQN framework in which a DDQN agent dynamically adjusts key PSO hyperparameters based on real-time constellation states, promoting an effective balance between global exploration and local exploitation during search.

## 3. Materials and Methods

This section details the complete methodology for our proposed adaptive constellation design approach. We begin by establishing a formal mathematical model of the optimization problem. This includes defining the foundational constellation architecture that constitutes the search space, as well as the key performance metrics. Subsequently, we introduce our novel solution: a hybrid framework that employs a DRL agent to intelligently control a Particle Swarm Optimizer.

### 3.1. Modeling and Problem Formulation

The primary step to effectively solve the constellation design task is to construct a precise mathematical model of the problem. In the context of LEO constellation design, this requires a rigorous definition of two fundamental elements: the solution space and the performance criteria. We introduced the foundational Walker constellation architecture, thereby defining the decision variables for our algorithm. Subsequently, the key system performance metrics such as GDOP and coverage multiplicity are introduced. These metrics are integrated into a multi-objective optimization function, which represents the core problem addressed by our algorithm. For navigation augmentation, the primary requirements are continuous coverage with a multiplicity greater than four and a minimal GDOP less than 10 to guarantee positioning accuracy [10,29]. GDOP, which reflects the quality of the satellite ground geometry, is a vital indicator of constellation performance. It is calculated as follows:

(1) $$\mathrm{GDOP}=\sqrt{\mathrm{trace}({(G^{T}G)}^{-1})}$$

where G is the Jacobian matrix whose elements are the partial derivatives of the satellite ground ranges with respect to user coordinates. $I_{x}^{\left(1\right)}\left(x_{k-1}\right)$ denotes the value of the partial derivative of the geometric distance from the receiver to the satellite with respect to *x* at $x_{k-1}$.

(2) $$G=\left[\begin{array}{cccc} -I_{x}^{\left(1\right)}\left(x_{k-1}\right) & -I_{y}^{\left(1\right)}\left(x_{k-1}\right) & -I_{z}^{\left(1\right)}\left(x_{k-1}\right) & 1\\ -I_{x}^{\left(2\right)}\left(x_{k-1}\right) & -I_{y}^{\left(2\right)}\left(x_{k-1}\right) & -I_{z}^{\left(2\right)}\left(x_{k-1}\right) & 1\\ \vdots & \vdots & \vdots & \vdots \\ -I_{x}^{\left(N\right)}\left(x_{k-1}\right) & -I_{y}^{\left(N\right)}\left(x_{k-1}\right) & -I_{z}^{\left(N\right)}\left(x_{k-1}\right) & 1 \end{array}\right]$$

**Figure 1:**
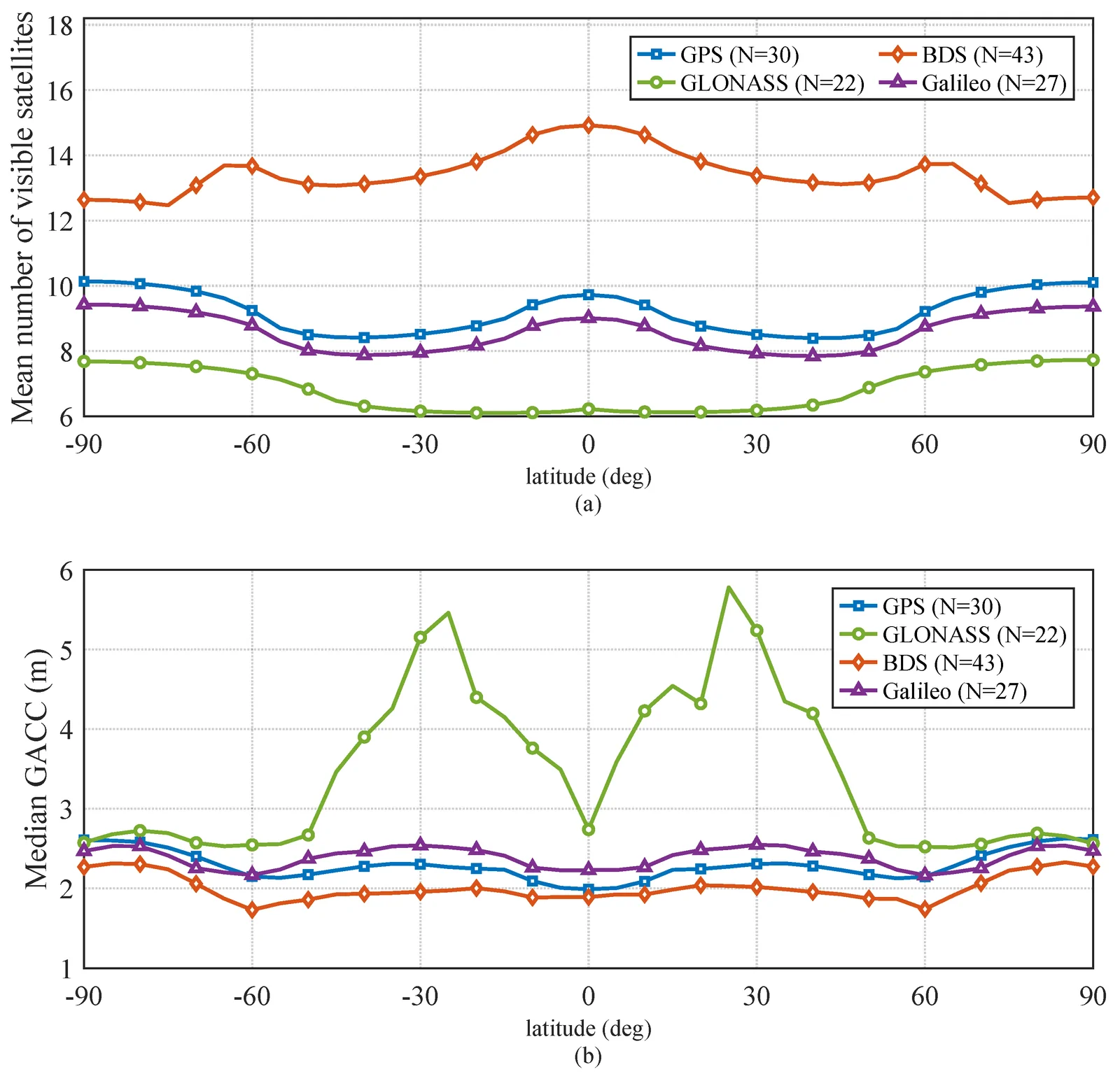
(**a**) Global coverage distribution of the GNSS constellation. (**b**) Navigation accuracy of the GNSS constellation.

GDOP objectives are widely adopted in LEO navigation augmentation constellation design to characterize geometry positioning quality [17,19,20]. We first analyze the performance distribution of existing Global Navigation Satellite Systems (GNSS). Figure 1 reveals the non-uniformity in its global coverage and navigation accuracy. Although GNSS provides a powerful global service, its configuration results in slightly poorer positioning performance at mid-to-low latitudes than at high latitudes [30].

The GLONASS performance in Figure 1 exhibits two GACC maxima near $-25^{\circ }$ and $25^{\circ }$. The elevated GACC is associated with poorly conditioned directional geometry rather than satellite numbers alone. The N = 22 satellite occupies the three nominal GLONASS orbital planes unevenly, which may strengthen the effect and explains why the two maxima are not exactly equal.

Based on the requirements for a regional navigation augmentation system, we formulate the constellation design as a multi-objective optimization problem [17]. The formulation is defined by the objective function to be minimized and the set of performance constraints that must be satisfied. The primary objectives are to achieve high performance with maximum resource efficiency. Therefore, the vector of objective functions F(X) is minimized consists of two competing goals:

1. minimizing the total number of satellites;
2. minimizing the GDOP to maximize positioning accuracy;

This can be formulated as finding the optimal vector of orbital parameters *X* that minimizes a vector of objective functions F(X):

(3) $$\begin{array}{c} \min\;F\left(X\right)=\left(N_{total}\left(X\right),f_{\mathrm{GDOP}}\left(X\right)\right)\\ s.t.\left\{\begin{array}{c} f_{\mathrm{cov}}\left(X\right)\geq C_{\min}\\ f_{\mathrm{continue}}\left(X\right)=S_{\min}\\ f_{\mathrm{number}}\left(X\right)\leq N_{\max}\\ f_{\mathrm{GDOP}}\left(X\right)\leq G_{\max} \end{array}\right. \end{array}$$

where $N_{\max}$ is the maximum allowable total number of satellites and is set to 120 in this study. It defines the upper limit of the satellite resource budget. $f_{\mathrm{cov}}$ is the coverage multiplicity; $f_{\mathrm{continue}}$ is the Coverage Continuity, means the percentage of time; $f_{\mathrm{GDOP}}$ is GDOP value. For the specific application in this paper, we aim for continuous four-fold coverage of a specific region with high geometric quality. Therefore, we define the constraints as $N_{\min}=4$, $S_{\min}=100\%$, and $G_{\max}=10$. These parameters can be customized for different mission requirements.

#### 3.1.1. Foundational Constellation Architecture

For this study, we selected the Walker-Delta constellation as the foundational architecture due to its optimal balance of optimization feasibility and mission suitability [31]. Its primary advantage is its highly structured and symmetric nature, which allows the entire constellation to be defined by a minimal set of parameters. This transforms the design task into a low-dimensional optimization problem. Therefore, the Walker framework allows our algorithm to focus on finding optimal parameter combinations within a stable search space.

#### 3.1.2. Orbital Parameter Mode

**Figure 2:**
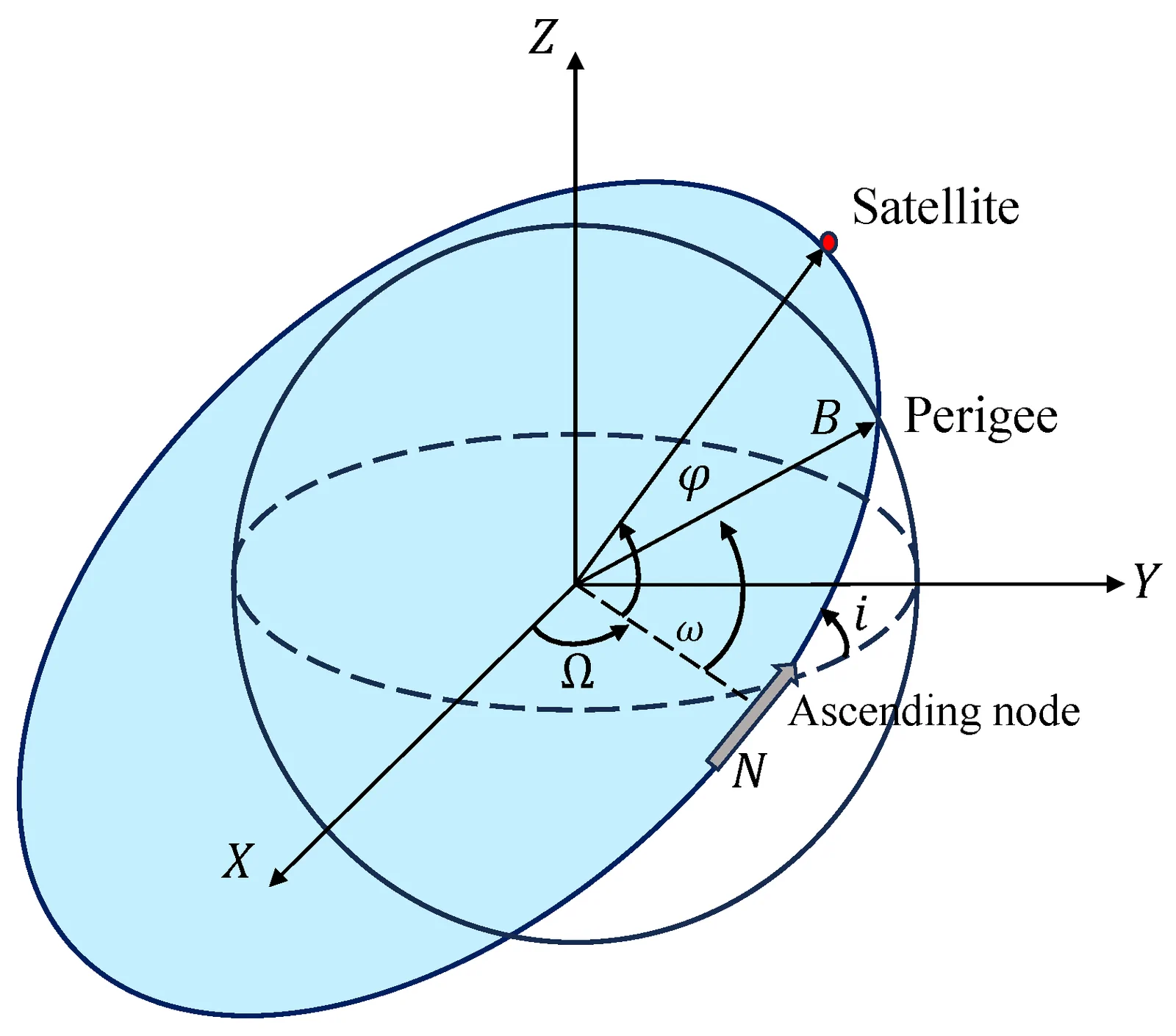
Illustration of the satellite orbital parameters.

The state of a satellite in a three-dimensional orbit is uniquely defined by six orbit elements. The orbit is given by the semi-major axis *a* and eccentricity *e*, and its plane’s orientation in space is defined by the inclination *i*, the argument of perigee *w*, and the right ascension of the Ascending Node (RAAN) Ω. The satellite’s specific in-orbit position is given by the true anomaly φ, derived from the mean anomaly *M* as illustrated in Figure 2. For practical reasons such as maintaining stable ground power levels, LEO missions commonly employ circular orbits. To establish a tractable optimization problem, we therefore simplify the model by making several key assumptions. We presuppose a circular orbit, setting the eccentricity e=0 and the semi-major axis to a constant value of a=7471 km. Furthermore, we assume that the satellites are uniformly distributed along this orbit. These assumptions constitute the orbital model defined in Equations (4)–(6).

(4) $$\dot{\Omega }\left(a,i\right)=-\frac{3}{2}J_{2}\frac{R_{E}^{2}}{a^{7/2}}\sqrt{\mu_{E}}\cos i$$

(5) $$\dot{\omega }\left(a,i\right)=\frac{3}{2}J_{2}\frac{R_{E}^{2}}{a^{7/2}}\sqrt{\mu_{E}}\left(2-\frac{5}{2}\sin^{2}i\right)$$

(6) $$\dot{M}\left(a,i\right)=\frac{3}{2}J_{2}\frac{R_{E}^{2}}{a^{7/2}}\sqrt{\mu_{E}}\left(1-\frac{3}{2}\sin^{2}i\right)+\sqrt{\frac{\mu_{E}}{a^{3}}}$$

Here, $\mu_{E}=GM_{E}$ denotes the standard gravitational parameter of the Earth, where *G* is the universal gravitational constant and $M_{E}$ is the mass of the Earth. Our orbital model accounts for the long-term secular drifts induced by the J2 perturbation to ensure fidelity. The argument of perigee is undefined for the circular orbits used in our study and is therefore excluded from our model.

#### 3.1.3. Satellite-to-Ground Coverage Model

**Figure 3:**
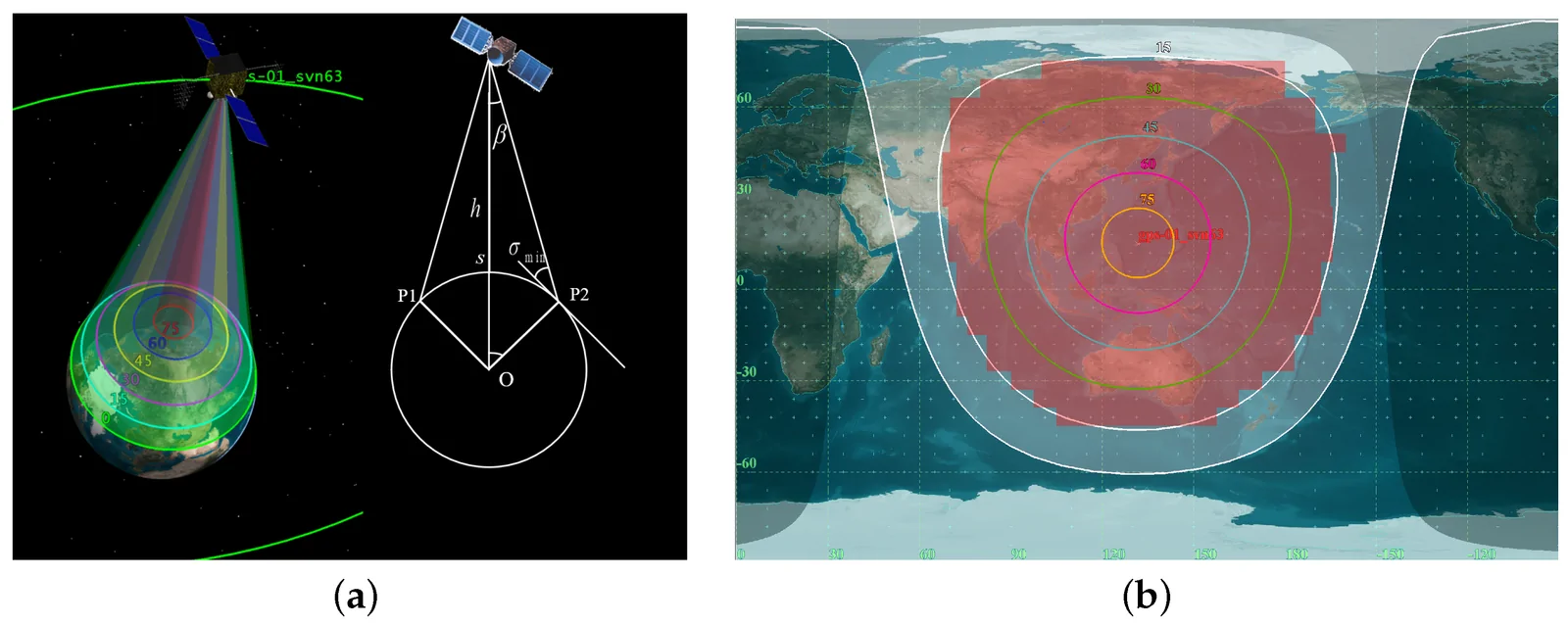
(**a**) The geometric model defining the coverage footprint. (**b**) Visualization of how the coverage footprint of a GPS satellite varies with the elevation angle.

The satellite’s minimum elevation angle, which dictates its effective coverage area, is a critical parameter for navigation. Since low elevation angles can result in signal blockage from terrain. Followed common practice for navigation tasks, we have set the minimum elevation angle at 7.5° for our simulations [32]. The inverse relationship between elevation angle and coverage area is illustrated in Figure 3, which shows the expanding footprint of GPS satellite SVN64 as the angle is reduced.

Let $R_{e}$ denote the Earth’s radius and *O* denote its center. For a satellite orbiting at an altitude h with a sub-satellite point s, we define the minimum observation angle from a ground target as the elevation angle $\sigma_{\min}$. The instantaneous coverage area of this satellite corresponds to a geocentric half-angle d. Based on geometric relationship between these variables, the geocentric half-angle can be derived as:

(7) $$d=\arccos\left(\frac{R_{e}\cos\sigma_{\min}}{R_{e}+h}\right)-\sigma_{\min}$$

The instantaneous coverage area $A_{s}$ of the satellite can be calculated as:

(8) $$A_{s}=4\pi {R_{e}}^{2}\sin^{2}\frac{d}{2}$$

Furthermore, from the spatial geometric relationship, the satellite’s instantaneous field of view β can be calculated as:

(9) $$\beta =\arcsin\left(\frac{R_{e}\cos\sigma_{\min}}{R_{e}+h}\right)=90^{\circ }-d-\sigma_{\min}$$

For a single satellite operating at a specific altitude, its coverage is constrained by both the Earth’s curvature and the user terminal’s minimum elevation angle. This results in the satellite forming an instantaneous circular coverage area on the Earth’s surface.

However, transitioning from a single-satellite model to an analysis of the overall performance of a multi-satellite constellation is not a simple linear superposition. This complexity arises from several factors. First, the instantaneous coverage footprint of the constellation and its coverage multiplicity are constantly changing over time. Second, the relative geometry among the satellites continuously evolves, creating dynamically shifting regions of overlapping coverage and coverage gaps. Finally, the constellation’s aggregate performance is a function of the total number of satellites, the number of orbital planes, the orbital inclination and altitude, and the inter-satellite phasing relationships.

Therefore, the core challenge of constellation design shifts from analyzing a single satellite’s coverage to searching within this variable space for an optimal combination of parameters. The goal is to satisfy specific performance metrics while utilizing the fewest possible satellite resources. This non-linear problem needs the advanced intelligent optimization algorithms for an effective solution. It motivates the introduction of our reinforcement learning framework for the adaptive control of a metaheuristic algorithm.

#### 3.1.4. Hybrid Deep Reinforcement Learning Metaheuristic Framework

In order to overcome the reliance of conventional metaheuristic algorithms on static hyperparameters, we proposed a hybrid optimization framework based on deep reinforcement learning [27,28]. The framework is designed to enable adaptive control over the core behaviors of a metaheuristic algorithm through a learning process. Its central idea is to model the iterative optimization process of the metaheuristic as a Markov Decision Process (MDP) and to train a DRL agent to act as a controller.

To enable adaptive hyperparameter control, we cast the PSO self-tuning process as a Markov decision process and define the agent interface in terms of state, action, and reward.

- State: In iteration *t*, the agent observes a macroscopic description of the swarm and current best solution,
  (10) $$s_{t}={\left[P\left(t\right),\;C\left(t\right),\;S\left(t\right),\;D\left(t\right),\;G\left(t\right)\right]}^{T},$$
  where P(t), C(t), and S(t) denote the GDOP, coverage multiplicity, and satellite number of the current best constellation, respectively. D(t)∈[0, 1] is the normalized population diversity that characterizes exploration versus convergence,
  (11) $$D\left(t\right)=\frac{1}{N_{p}L_{D}}\sum_{i=1}^{N_{p}}\sqrt{\sum_{d=1}^{D}{\left(\frac{x_{i}^{\left(d\right)}\left(t\right)-{\bar{x}}^{\left(d\right)}\left(t\right)}{x_{d,\max}-x_{d,\min}}\right)}^{2}},$$
  and G(t)∈[−1, 1] is the normalized fitness gain over a sliding window of size *k*,
  (12) $$G\left(t\right)=\tanh\;\left(\frac{J\left(p_{g}\left(t\right)\right)-J\left(p_{g}\left(t-k\right)\right)}{J(p_{g}\left(t-k\right))}\right).$$
- Action: The action $a_{t}$ selects the PSO hyperparameter setting for the next iteration. We discretize the continuous space into *K* predefined strategies,
  (13) $$A=\left\{a_{1},\;a_{2},\ldots ,\;a_{K}\right\},\;a_{k}=\left[w_{k},\;c_{1,k},\;c_{2,k}\right],$$
  where $w_{k}$, $c_{1,k}$, and $c_{2,k}$ correspond to inertia, cognitive, and social coefficients, respectively.
- Transition: After the agent selects $a_{t}$, the swarm evolves for one PSO iteration under the chosen hyperparameters, and the next state $s_{t+1}$ is computed deterministically from the updated population statistics.
- Reward: The reward encourages progress while maintaining exploration when improvement stalls,
  (14) $$R\left(s_{t},\;a_{t},\;s_{t+1}\right)=\left\{\begin{array}{cc} R_{p}, & J\left(p_{g}\left(t\right)\right)>J\left(p_{g}\left(t-1\right)\right),\\ R_{s}, & J\left(p_{g}\left(t\right)\right)=J\left(p_{g}\left(t-1\right)\right)\;\mathrm{and}\;D\left(t\right)>D_{\mathrm{th}},\\ R_{n}, & \mathrm{otherwise}. \end{array}\right.$$
  Here, $R_{p}$ rewards improvement, $R_{n}$ penalizes stagnation, and $R_{s}$ promotes exploration when the swarm remains diverse. The window length *k* and diversity threshold $D_{\mathrm{th}}$ are algorithmic hyperparameters.

We set k=5, so that the recent progress indicator H(t) compares the current global best cost with that obtained five iterations earlier. It reduces sensitivity to single iteration fluctuations while allowing the controller to respond promptly to changes in search progress.

The diversity measure D(t) is bounded between 0 and 1 because each decision variable is normalized by its search range. We set $D_{\mathrm{th}}=0.10$, treating a mean normalized root mean square (RMS) distance from the swarm centroid greater than 0.10 as evidence that meaningful population diversity remains. Accordingly, temporary stagnation above this threshold receives the exploration reward $R_{s}$, whereas low diversity stagnation receives the penalty $R_{n}$.

This MDP formulation allows the DDQN agent to learn a hyperparameter control policy for PSO, improving the balance between exploration and exploitation in constrained constellation optimization.

#### 3.1.5. The PSO-DDQN Algorithm

**Figure 4:**
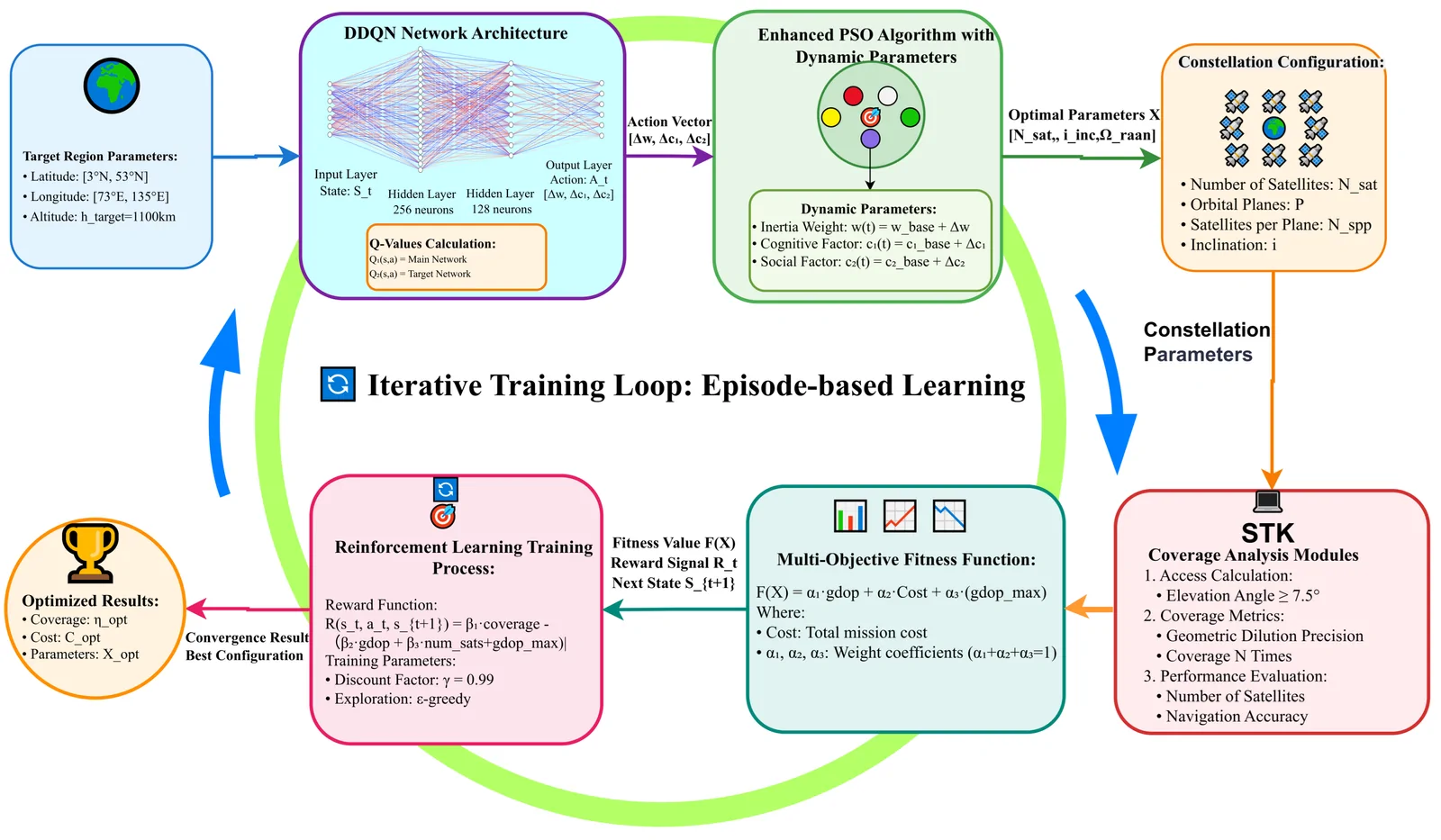
Block diagram of PSO-DDQN algorithm.

For the efficient optimization of regional Walker constellation coverage, we propose a novel hybrid intelligence algorithm, PSO-DDQN, which pairs a Double Deep Q-Network with an PSO algorithm. Figure 4 presents a detailed block diagram of the PSO-DDQN algorithm. The figure illustrates the complete information flow of the iterative training loop: from the constellation performance evaluation in the STK environment, to the state representation and decision-making within the DDQN agent, and finally to the agent’s adaptive control over the PSO’s hyperparameters. This process allows the agent to learn an effective control policy directly from the optimization task.

PSO-DDQN utilizes PSO as the base search algorithm, governed by a DDQN agent acting as a controller. This agent learns to dynamically modulate PSO’s key hyperparameters (inertia weight *w*, cognitive factor $c_{1}$, and social factor $c_{2}$), thereby improving the algorithm’s exploration-exploitation balance and overall convergence.

The synergy between DDQN and PSO is realized through a hierarchical, two-stage decision architecture. In the high-level stage, the DDQN agent learns the long-term value of constellation states. Its state space, *S*, is represented by a feature matrix capturing essential configuration properties like orbital element distributions, coverage metrics, and link topology. The DDQN’s objective is to approximate the optimal action-value function $Q^{*}\left(s,a\right)$, which predicts the maximum expected return of performing action in state. In the low-level stage, the PSO module acts as a dedicated action-solver. Rather than selecting from a discrete set of actions, the DDQN agent delegates the action-finding task to the PSO. The PSO then performs a localized search in the continuous action space, where each particle is a candidate action vector. The fitness of each particle-action is determined by the DDQN’s learned Q-value. Therefore, the PSO’s objective is to find the action a that maximizes the Q-function for the current state. This mechanism ingeniously converts discrete action-selection problem of standard DRL into a continuous optimization sub-problem, solved efficiently by PSO.

The objective of our optimization is to identify a set of Walker constellation parameters that yields optimal coverage performance for a designated region. The decision variable vector X is defined as:

(15) X=[P, S, F, I]

where *P* is the number of orbital planes, *S* is the number of satellites per orbital plane, and *F* is the phasing factor between adjacent orbital planes, satisfying 0≤F≤P−1. In Walker-Delta notation (T/P/F), where T is the total number of satellites, F is a dimensionless integer that determines the relative angular displacement along the track between corresponding satellites in adjacent orbital planes. The variable *I* represents the common orbital inclination for all orbits.

The initial motivation of our study was to achieve optimal coverage of the target region at a fixed orbital altitude. This is because the selection of an orbital altitude is subject to numerous practical constraints. Our navigation augmentation system was originally designed to be hosted on existing Low Earth Orbit (LEO) satellite platforms, rather than requiring the launch of an independent satellite constellation. Consequently, we did not include orbital altitude as an optimization parameter.

**Figure 5:**
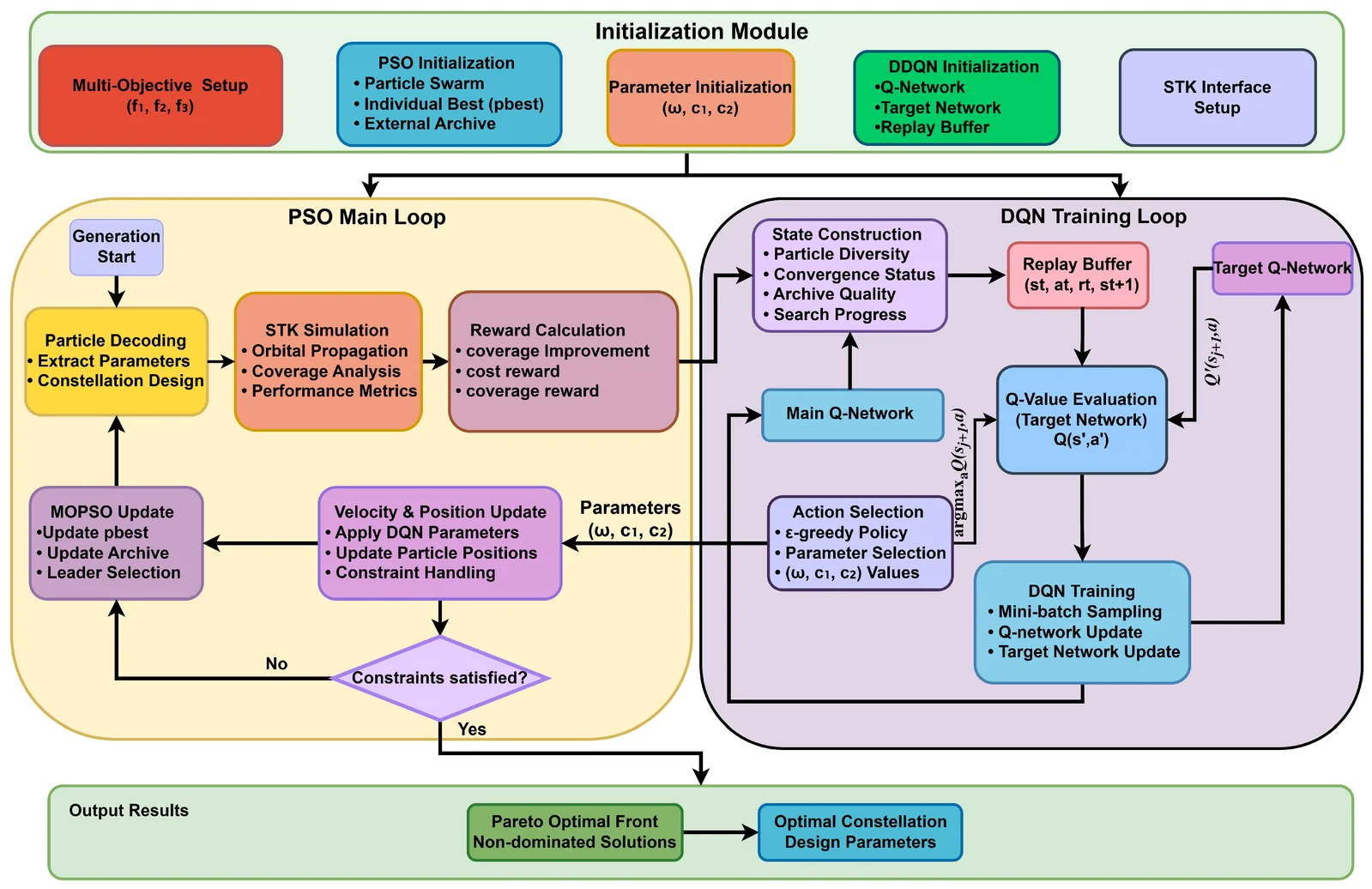
LEO-NA constellations design problem.

- Foundational Algorithm: Particle Swarm Optimization;
  Particle Swarm Optimization (PSO) is a classic metaheuristic inspired by population evolution, where each particle, representing a candidate solution, moves through the search space based on its own experience and that of the entire swarm. For the D-dimensional constellation design problem, a particle’s position vector $x_{i}=\left(x_{i1},\;x_{i2},\cdots ,\;x_{iN}\right)$ encodes a set of design parameters, and its velocity vector $v_{i}=\left(v_{i1},\;v_{i2},\cdots ,\;v_{iN}\right)$ dictates its movement. The dynamics of PSO are governed by the velocity and position update equations:
  (16) $$\begin{array}{c} v_{i}\left(n+1\right)=w\times v_{i}\left(n\right)+c_{1}\times r_{1}\times \left(p_{i}\left(n\right)-x_{i}\left(n\right)\right)\\ +c_{2}\times r_{2}\times \left(p_{g}\left(n\right)-x_{i}\left(n\right)\right) \end{array}$$
  The position update equation is
  (17) $$x_{i}\left(n+1\right)=x_{i}\left(n\right)+v_{i}\left(n+1\right)$$
  In these equations, *n* is the iteration, *w* is the inertia weight, $c_{1}$ and $c_{2}$ are the cognitive and social coefficients, while $p_{i}\left(n\right)$ and $p_{g}\left(n\right)$ are the personal and global best positions found so far. The algorithm’s behavior is highly sensitive to its hyperparameters [33]. The acceleration coefficients are crucial as they balance the influence of individual versus collective knowledge. However, they are typically set empirically, limiting the algorithm’s adaptability across different problems. Ideally, the search should be more explorative initially and become more exploitative in later stages as the swarm converges on a solution. This exploration–exploitation trade-off is primarily controlled by the inertia weight. A large ω promotes global exploration by allowing particles to maintain momentum and traverse the search space, thus avoiding premature convergence. A small ω, in contrast, promotes exploitation by dampening movement, enabling fine-grained searches within promising regions to improve solution accuracy.
- The DDQN-based Controller for PSO;
  The Double Deep Q-Network (DDQN) algorithm learns an optimal policy, $\pi^{*}:S\to A$, to maximize cumulative discounted rewards by interacting with an environment. The core of this method is the state-action value function $Q^{*}\left(S_{t},\;A_{t}\right)$, which represents the expected return from taking action $A_{t}$ in state $S_{t}$. This function is learned iteratively through updates based on environmental feedback.
  To handle complex problems, DDQN approximates the Q-function with a deep neural network. Its key innovation over standard DQN is its dual-network architecture, designed to combat the systematic overestimation of Q-values. This architecture consists of:
  (18) qS(s, a)=.Gt∣S0=s, A0=aS
  The agent iteratively updates the value function $Q^{*}\left(S_{t},\;A_{t}\right)$ based on experience sampled from the environment:
  (19) $$Q^{*}\left(S_{t},\;A_{t}\right)=Q\left(S_{t},\;A_{t}\right)+\alpha \left[R_{t+1}+\gamma \max_{a^{\prime }\in A}Q\left(S_{t+1},\;a^{\prime }\right)-Q\left(S_{t},\;A_{t}\right)\right]$$
  where *t* is the current time step, α is the learning rate, γ is the value discount factor, $\max_{a^{\prime }\in A}Q\left(S_{t+1},\;a^{\prime }\right)$ is the maximum Q-value achievable from the next state. DDQN utilizes a deep neural network $Q\left(s,\;a;\theta \right)$, to approximate the Q-function, $Q^{*}\left(S_{t},\;A_{t}\right)$. To address the problem of systematic Q-value overestimation found in standard DQN, DDQN employs a dual-network architecture: Online Network: This network is used to select the optimal action under the current policy. Target Network: This network is used to evaluate the value of the action selected by online network. Its parameters are periodically copied from online network, keeping them stable for a period. The online network’s parameters are trained by minimizing a loss function L(θ), typically the mean squared error between the predicted and target Q-values:
  (20) $$L\left(\theta \right)=E_{\left(s_{t},a_{t},r_{t},s_{t+1}\right)∼U\left(B\right)}\left[{\left(y_{t}^{\mathrm{DDQN}}-Q\left(s_{t},\;a_{t};\theta \right)\right)}^{2}\right]$$
  The online network chooses the best next action, but the target network provides its value. This mitigates overestimation and stabilizes the learning process. This entire process is depicted in Figure 5.
  The PSO explores the constellation design space, generating state information for DDQN. The DDQN, in its role as a meta-controller, processes this state and selects an action to adapt the PSO’s hyperparameters. The PSO then executes its search under this new policy, and the resulting change in performance is used to calculate a reward, closing the feedback loop. In the design of our PSO-DDQN algorithm, two choices are particularly noteworthy for their rationale:
  1. Action Space Discretization;
    We transform the continuous PSO hyperparameter space into a discrete set of actions. The primary benefit is that it reframes the difficult task of parameter tuning as a higher-level strategic selection, which substantially lowers the learning burden on the DDQN agent. The efficacy of this approach is borne out by our experimental results.
  2. DDQN Hyperparameter Configuration;
    While the DDQN agent introduces its own set of hyperparameters, we configured them based on widely accepted conventions in the RL community, with minor initial adjustments.

## 4. Experimental Results and Discussion

This chapter presents a comprehensive evaluation of the performance of the PSO-DDQN algorithm in the practical task of designing the LEO navigation augmentation constellation. We tasked the algorithm with generating optimal constellations under varying satellite fleet sizes, with the ultimate goal of achieving robust coverage using the minimum number of satellites.

The success of each designed constellation was judged against stringent performance criteria required for navigation augmentation: continuous coverage with a multiplicity of at least four and a GDOP value consistently below 10. All simulations were conducted using a circular baseline orbit at 1100 km altitude and 6° resolution of the ground grid. The key performance indicators analyzed for each solution include its GDOP characteristics, coverage multiplicity, and the resulting positioning accuracy in the target region.

### 4.1. Experimental Design and Setup

To evaluate the practical performance of the PSO-DDQN algorithm, we designed a case study focused on optimizing an LEO navigation augmentation constellation for a specific mission.

The primary objective is to design a regional coverage constellation that optimizes performance over the China region [10,20]. To benchmark our results, we used the existing CentiSpace LEO navigation augmentation system as a point of comparison. CentiSpace is an LEO navigation augmentation system that provides real-time decimeter-level positioning services by enhancing existing GNSS like BeiDou for a variety of users, from autonomous driving to public safety [6]. Its impressive performance is backed by a substantial constellation of 120 satellites arranged in a Walker configuration with a 55° inclination, distributed across 12 orbital planes with a phasing factor of 0, and its orbit altitude is 975 km [34]. This high-performance system serves as a robust benchmark for our optimization results. In this case study, the number of satellites in the optimized constellation was limited to 120 for comparison with the CentiSpace constellation, which has the same number of satellites.

**Figure 6:**
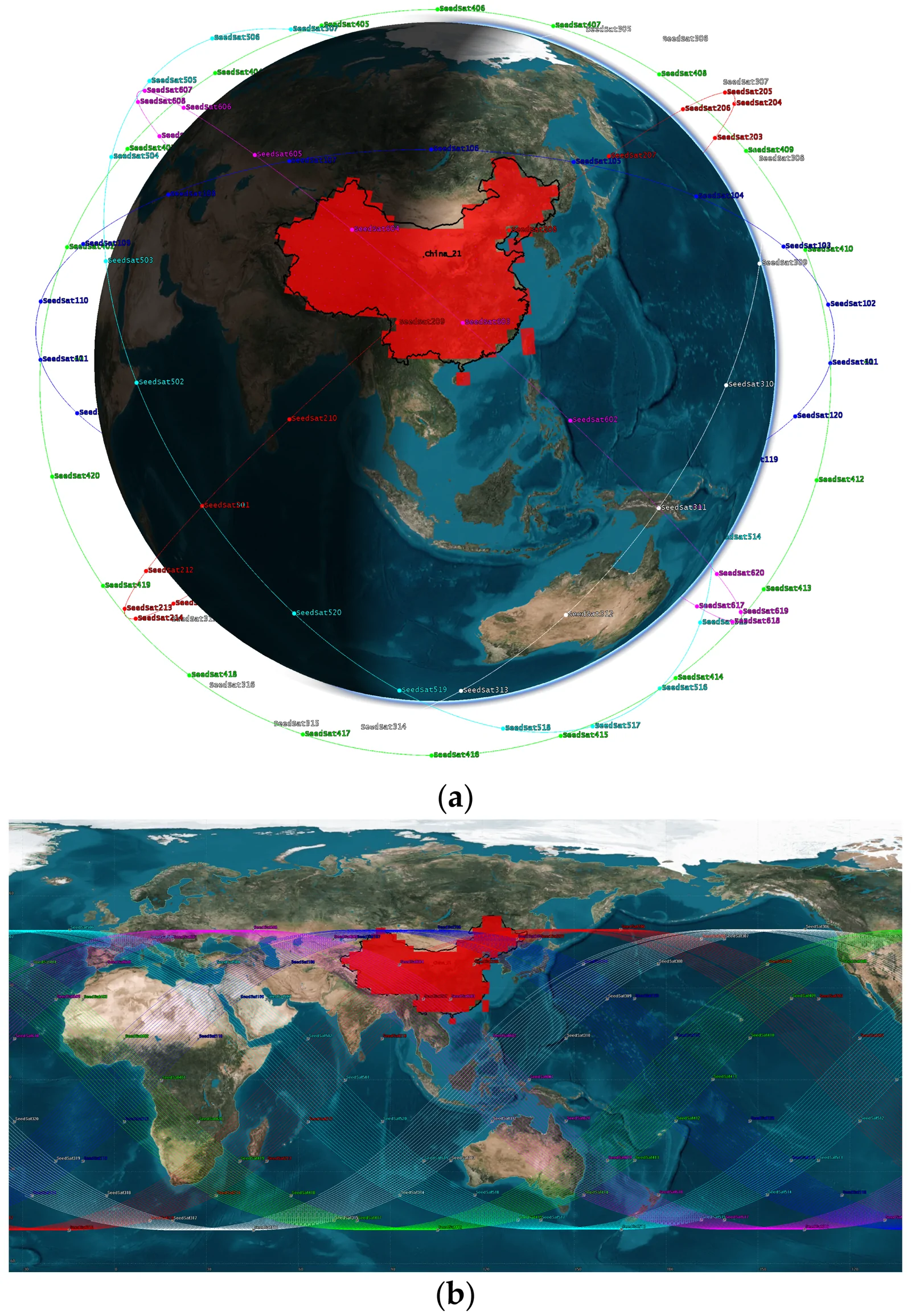
(**a**) Visualization of the optimized constellation for the target region. (**b**) Two-dimensional map of the corresponding satellite ground tracks over 24 h period.

The algorithmic workflow integrates PSO with the Satellite Tool Kit (STK). The PSO algorithm generates candidate Walker constellation parameters, which are passed to STK for coverage analysis. The satellite orbit simulations and algorithm implementations were conducted using STK (version 11.8, Analytical Graphics, Inc., Exton, PA, USA) and MATLAB (version 2024a, The MathWorks, Inc., Natick, MA, USA). All experiments and model training were executed on a workstation equipped with an AMD EPYC 9354 32-Core Processor (Advanced Micro Devices, Inc., Santa Clara, CA, USA), 384 GB of RAM, and an NVIDIA GeForce RTX 4090 GPU (NVIDIA Corp., Santa Clara, CA, USA). Figure 6 shows a representative optimal constellation designed by the PSO-DDQN algorithm.

The PSO-DDQN algorithm converged on a high-performance constellation parameters. Table 1 summarizes the detailed parameters of these optimal designs. A particularly noteworthy characteristic among these solutions is the algorithm’s consistent convergence towards high-inclination retrograde orbits.

### 4.2. Agent Training Dynamics

**Figure 7:**
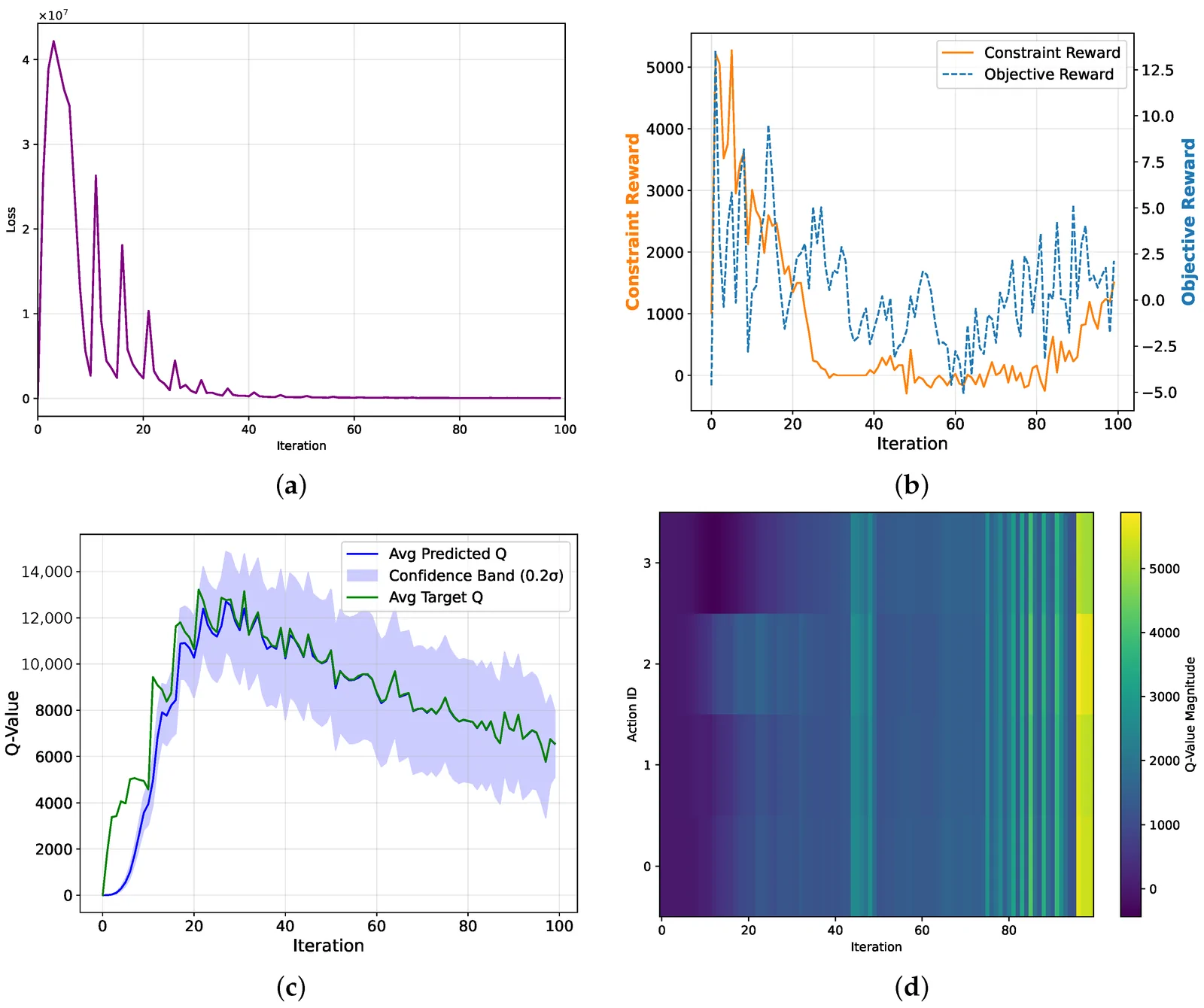
Training dynamics and policy evolution of the proposed PSO-DDQN method. (**a**) The convergence of TD loss during training. (**b**) The decomposed reward dynamics show the interaction between constraint-related reward and objective-related reward. (**c**) The evolution of predicted and target Q-values. (**d**) The Q-value evolution heatmap of the policy adaptation process.

Before evaluating the final constellation optimization performance of the PSO-DDQN algorithm, it is essential to analyze and validate the DDQN agent has itself undergone an effective learning process. To this end, we recorded a series of key internal metrics of the agent throughout its training and have summarized them in Figure 7.

This set of plots demonstrates the agent’s complete evolutionary path from initial random exploration to the formation of an effective control policy. As shown in Figure 7a, the TD loss rapidly decreases and stabilizes, indicating the convergence of value learning. Figure 7b further reveals that the temporary degradation of objective reward is mainly caused by the agent prioritizing constraint satisfaction, rather than learning instability. The evolution of predicted and target Q-values in Figure 7c confirms that value estimation remains stable despite environmental non-stationarity. Finally, Figure 7d illustrates the adaptive nature of the learned policy, where action preferences are continuously re-evaluated in response to dynamic changes and gradually stabilize in later training stages.

## 5. Results

### 5.1. GDOP Value Analysis

The optimized constellation parameters are shown in Table 1. A low and stable GDOP is a critical indicator of a robust constellation design, as it directly translates to higher positioning accuracy for users. A common requirement for PNT services is to maintain a GDOP value below 10 [10,17]. In Walker (T/P/F) notation, *T* denotes the total number of satellites, *P* denotes the number of orbital planes, and *F* is the Walker phasing factor that determines the relative offset along the trajectory between satellites in adjacent orbital planes.

**Figure 8:**
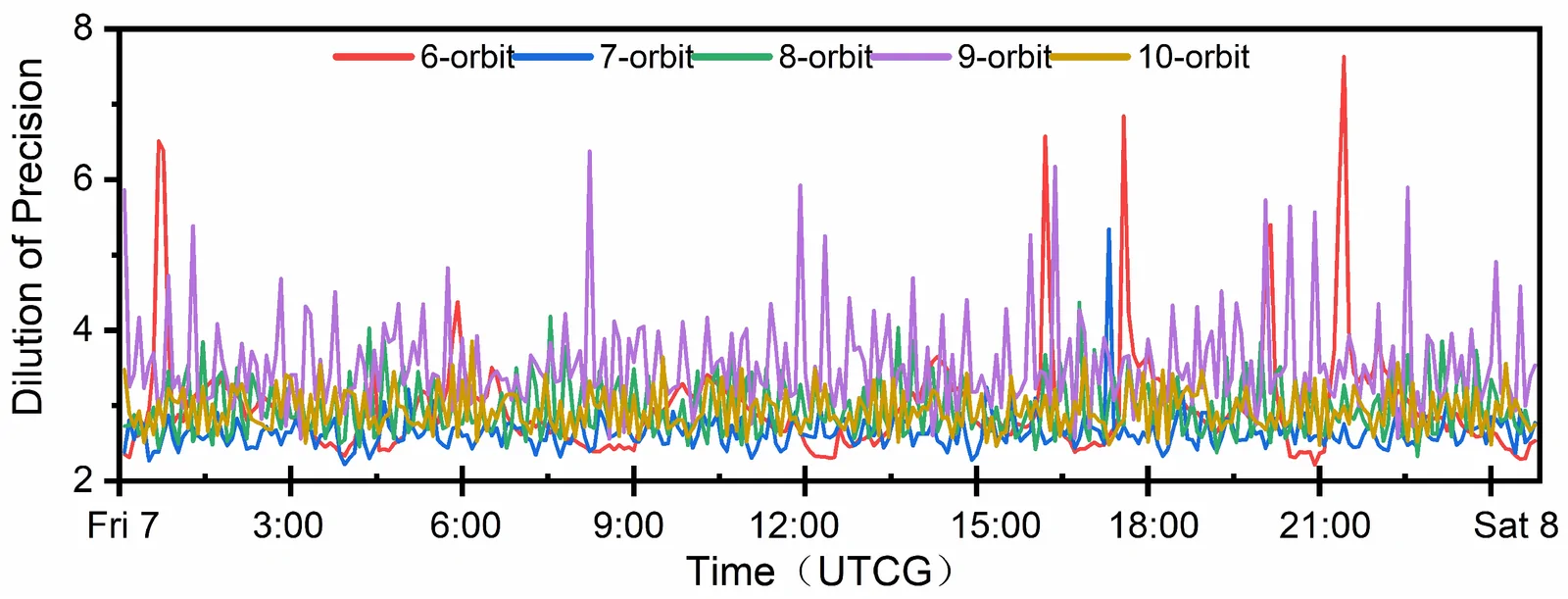
Comparison of dilution of precision of optimized constellation.

**Figure 9:**
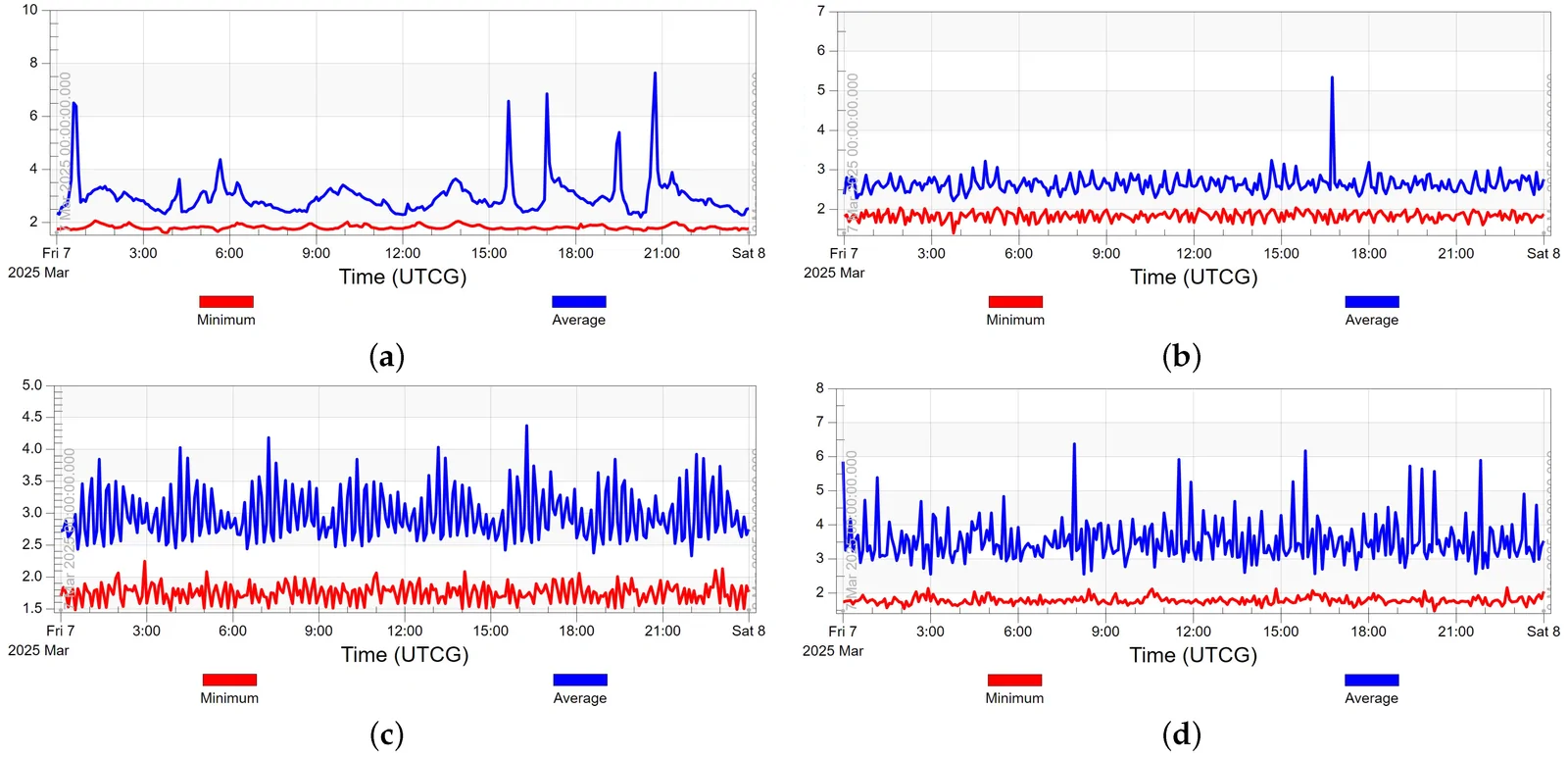

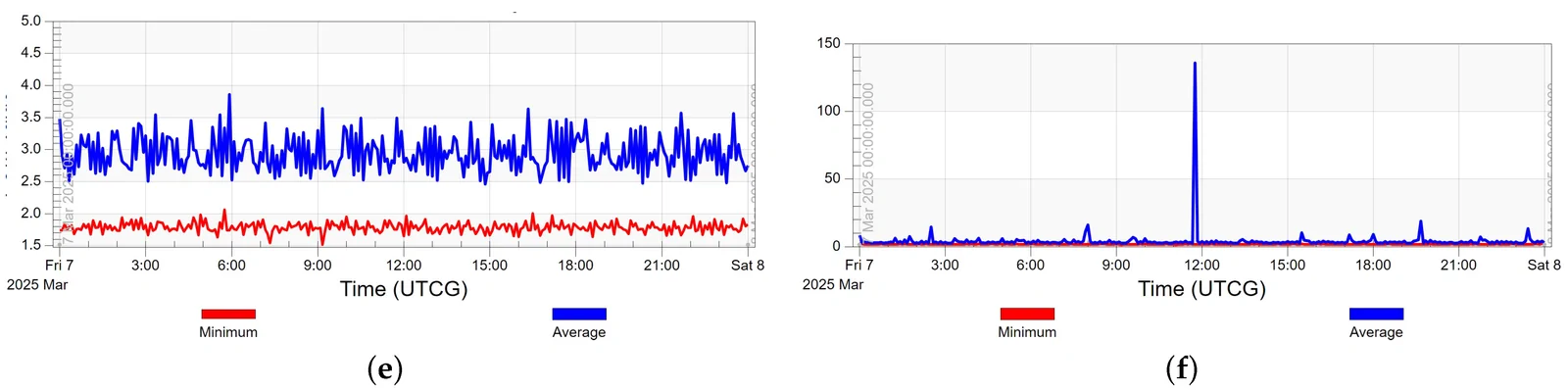
GDOP Comparison of optimized constellation and Benchmark constellation (**a**) GDOP of 6-orbit constellation. (**b**) GDOP of 7-orbit constellation. (**c**) GDOP of 8-orbit constellation. (**d**) GDOP of 9-orbit constellation. (**e**) GDOP of 10-orbit constellation. (**f**) GDOP of CentiSpace constellation.

Figure 8 evaluates the 24 h GDOP performance of several constellations designed by our algorithm, each with a different number of orbital planes. The optimized constellations exhibit favorable geometric properties, with average GDOP values consistently below 3 and maximum values remaining under 8 throughout the simulation period, thereby satisfying the design requirements. For instance, the 10-plane configuration maintains GDOP values below 4 across the entire day. In contrast, the CentiSpace benchmark constellation (Figure 9f) exhibits pronounced GDOP fluctuations, with multiple sharp peaks over time. Such instability could degrade the reliability of navigation services, underscoring the superior geometric robustness achieved by the proposed PSO-DDQN based designs.

### 5.2. Coverage Multiplicity Analysis

**Figure 10:**
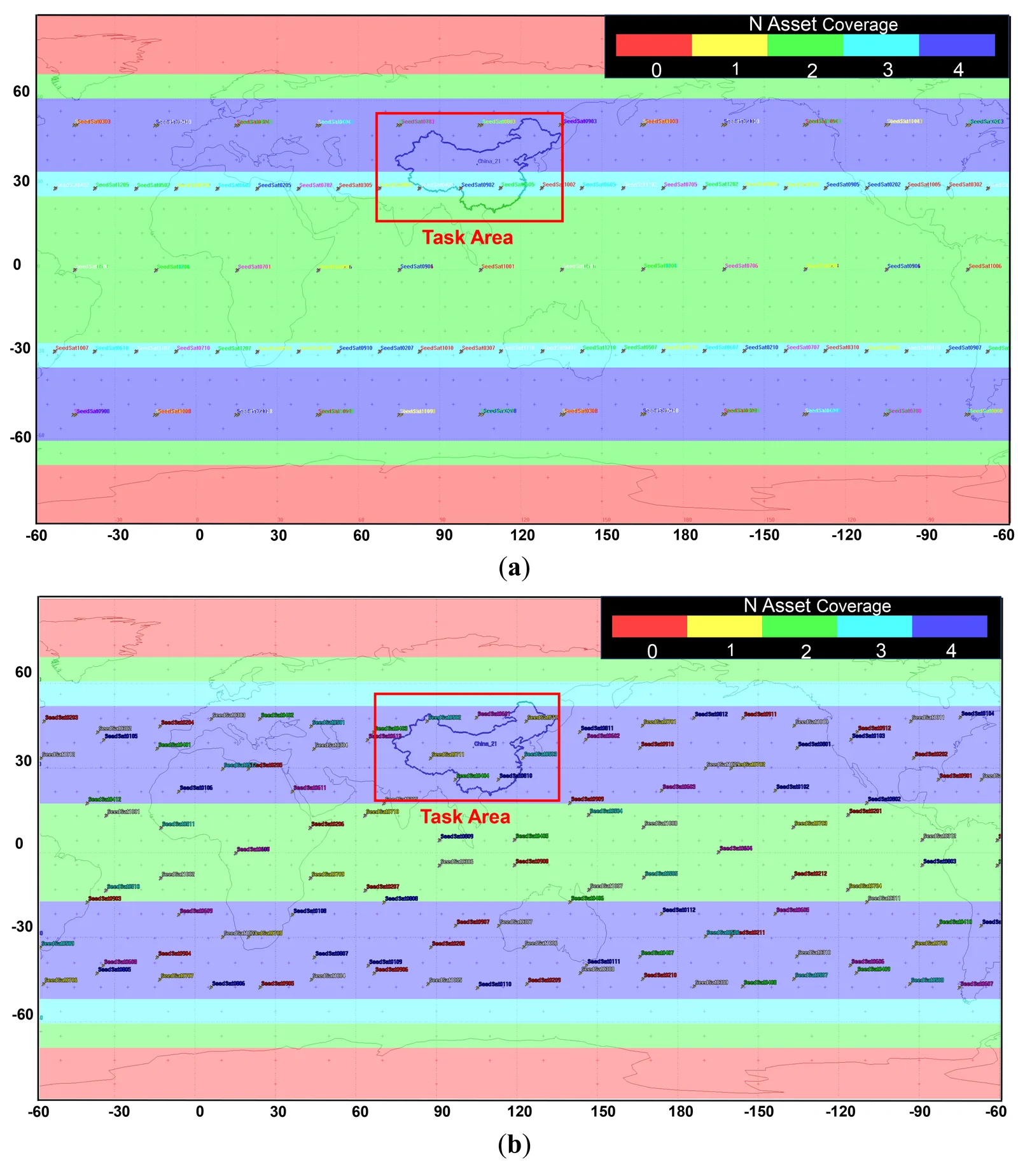
Comparative analysis of coverage multiplicity. (**a**) The benchmark constellation, showing insufficient 2-fold coverage over the target region. (**b**) The optimized constellation, demonstrating consistent 4-fold coverage.

Coverage multiplicity is a critical parameter in assessing whether a LEO navigation augmentation system can ensure continuous PNT service availability in a given region. Since at least four satellites are required for navigation and positioning, a fundamental requirement for such a system is to ensure that at least four satellites are visible from the target region at all times of the day. A constellation that meets this requirement also possesses the capability for independent navigation. The 24 h variation of coverage multiplicity over the China region for our optimized constellations is illustrated in Figure 10.

Figure 10 provides a comparison of the coverage multiplicity performance between the optimized constellation and the benchmark constellation over the target region. The benchmark constellation provides only 2-fold coverage (light blue areas) over a significant portion of the target region (China), which is insufficient to meet the fundamental 4-satellite requirement for navigation services. In contrast, the bottom panel shows that the optimized constellation achieves consistent 4-fold (purple areas) coverage across the vast majority of the same region. This improvement ensures continuous and independent PNT service.

At each epoch, the minimum, mean, and maximum GDOP values were calculated across all grid points within the target region. The values reported in Table 2 are the temporal means of these three regional statistics over the 289 epochs of the 24 h analysis period. Regional GDOP performance was evaluated using 20 stationary ground points distributed over the target region in China. The evaluation region extends from 21° N to 55° N and from 81° E to 124° E, with a grid spacing of 6°. At each 5 min epoch, GDOP was calculated independently for every ground point.

This type of non-intuitive design strategy is seldom employed in conventional constellation design. Its discovery here fully demonstrates the powerful global search capability of the PSO-DDQN algorithm. It highlights the algorithm’s ability to break through the limitations of conventional design wisdom, finding superior parameters in a complex space to achieve the goal of high efficiency with fewer satellites.

### 5.3. PPP Analysis

We conducted an additional GREAT-LAG simulation at the WUH2 station. Table 3 summarize the PPP experiment. Both 120-satellite LEO augmentations substantially shortened convergence relative to GPS only: the median $t_{0.30}$ was 21.25 min for GPS only, 2.25 min for the reference constellation, and 2.00 min for the optimized constellation. For the stricter 0.10 m threshold, GPS only reached convergence in three of four arcs with a median of 76.50 min among the reached arcs, whereas both LEO-augmented cases reached it in all four arcs, with medians of 3.50 and 3.00 min for the reference and optimized constellations, respectively.

**Figure 11:**
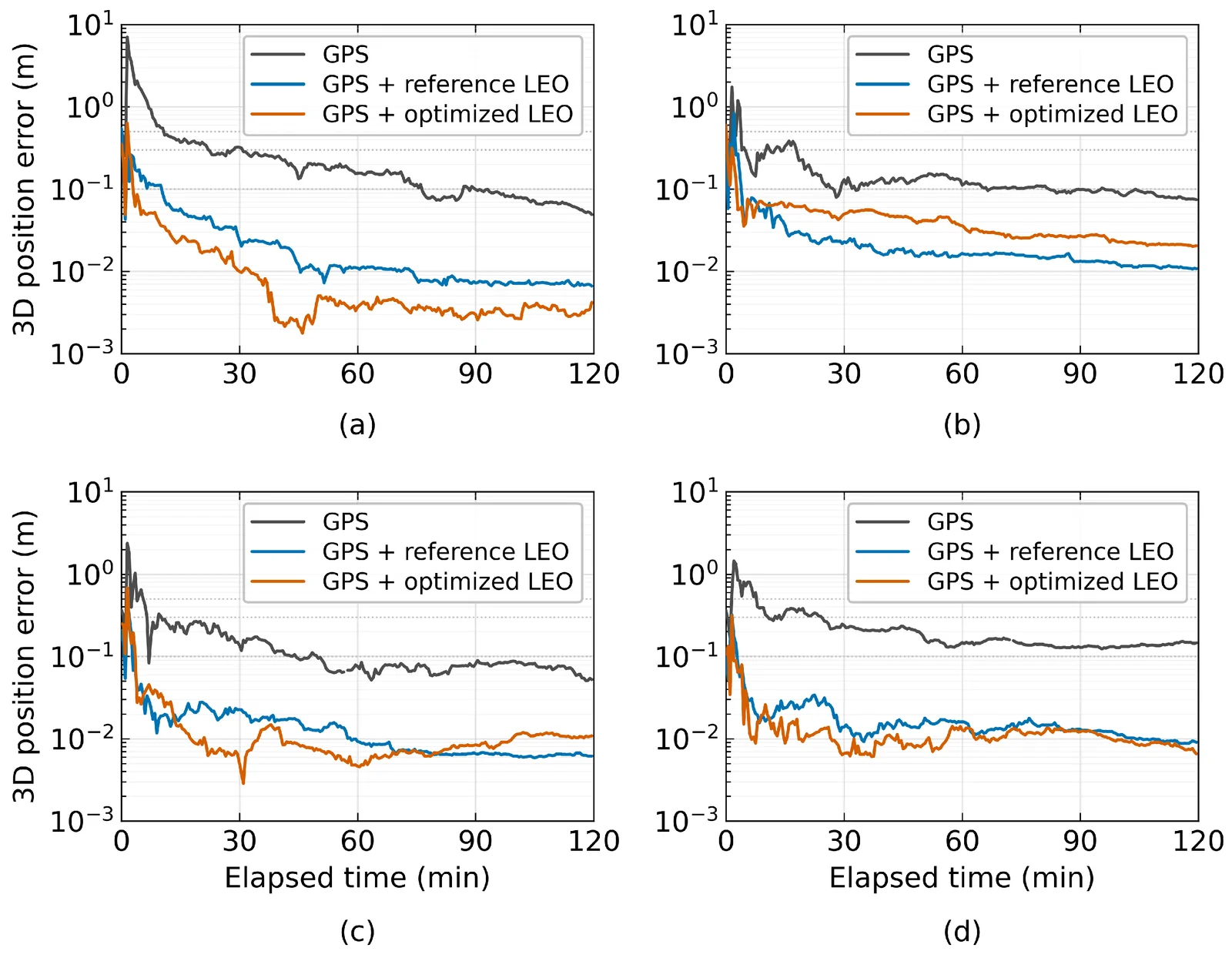
Three-dimensional PPP position errors for GPS only, GPS augmented by the reference constellation, and GPS augmented by the optimized constellation in four non-overlapping 2 h arcs at WUH2. (**a**) Error convergence during the first arc from 00:30 to 02:30; (**b**) Error convergence during the second arc from 06:30–08:30; (**c**) Error convergence during the third arc from 12:30–14:30; (**d**) Error convergence during the forth arc from 18:30–20:30.

The optimized constellation used more satellites than the reference in the paired comparison. For $t_{0.30}$, it was one 30 s epoch faster in two arcs and tied in two; for $t_{0.10}$, it was faster in three arcs and slower in one, with a paired median difference of −1.00 min. The RMSE was lower for the optimized case in three of four arcs, with a paired median difference of −6.66 mm; As shown in Figure 11, compared to GPS only, both LEO-assisted scenarios produced a substantial improvement in this controlled simulation. Across the four arcs, the median 0.30 m convergence time decreased from 21.25 min for GPS only to 2.25 min for the reference constellation and 2.00 min for the optimized constellation.

## 6. Discussion

Our study addresses the constrained multi-objective problem of regional LEO navigation constellation design. The optimized constellation demonstrates substantial improvements in spatial coverage uniformity and GDOP stability in the target region. By replacing static heuristic parameter settings with adaptive control, the optimization process converges more consistently toward geometrically balanced orbital configurations. As evidenced by the policy heatmap (Figure 7d), the reinforcement learning agent developed a dynamic strategy that balances exploration. These results indicate that intelligent optimization strategies can improve the effectiveness of regional augmentation constellation deployment.

The optimization process exhibited two distinguishable stages, which are distinctly reflected in the decomposed reward dynamics in Figure 7b. In the initial phase, candidate constellations frequently violated coverage constraints, and the search trajectory was primarily guided toward feasibility. Once stable feasible configurations were achieved, the optimization shifted toward improving geometric quality, particularly through GDOP minimization. By stabilizing the search within the feasible region before pursuing geometric optimization, the framework avoids convergence toward mathematically optimal yet operationally infeasible solutions.

Increasing the maximum allowable constellation size expands the number of candidate configurations and reduces the fraction of the search space that can be explored. This may increase the risk of premature convergence to identify a feasible solution. Moreover, the computational cost of STK evaluations increases with the number of satellites. Therefore, the present results are limited to the constellation configurations investigated in this study. Applying the proposed method to larger constellation design problems would require independent validation and retraining of the controller.

## 7. Conclusions

The design of LEO constellations presents a formidable optimization challenge, where traditional metaheuristics are often hampered by their reliance on static hyperparameters, leading to suboptimal solutions. This paper introduced a new paradigm to dismantle this bottleneck: a hybrid PSO-DDQN framework where a deep reinforcement learning agent acts as an intelligent meta-controller for a Particle Swarm Optimizer.

Our central innovation was to reframe the parameter control problem as a learning task. The DDQN agent, guided by a carefully engineered MDP, learns a dynamic control policy from its interaction with the PSO search process. This enables it to masterfully balance the exploration–exploitation trade-off in a way that fixed rules cannot.

The efficacy of this approach was unequivocally demonstrated through a demanding regional constellation design task. The PSO-DDQN algorithm not only outperformed all benchmark heuristics in both convergence speed and final solution quality but also discovered a constellation design superior to the CentiSpace system, achieving a 27% improvement in average GDOP. The discovery of non-obvious, high-performance strategies, such as the strategic use of retrograde orbits, is a testament to the framework’s powerful global search capabilities and its capacity to transcend conventional design heuristics. This work establishes the DRL-metaheuristic approach as an effective method for solving complex aerospace optimization problems. It paves the way for a new generation of intelligent, self-tuning optimizers that can achieve high efficiency with fewer resources. Future research will aim to enhance the framework’s control precision by exploring continuous action spaces and to test its robustness on dynamic and multi-objective optimization problems across various engineering domains.

## Figures and Tables

**Table 1:** Parameters of the optimized constellations.

Planes	Number of Satellites	Factor	Inclinaton
6	120	0	131.19
7	119	1	131.47
8	120	1	134.44
9	117	4	129.4
10	120	6	47.79

**Table 2:** Performance comparison of optimized and benchmark constellations.

Planes	Satellites	GDOP			Coverage		
		Min	Avg	Max	Min	Avg	Max
6	120	1.82	2.97	9.64	5.12	7.22	8.66
7	119	1.84	2.64	5.12	5.40	7.16	8.91
8	120	1.75	2.97	5.24	5.57	7.18	8.48
9	117	1.79	3.54	12.62	5.61	7.95	9.80
10	120	1.79	2.94	6.17	5.50	7.21	9.00
CentiSpace	120	1.75	4.15	28.82	4.60	6.98	8.82

**Table 3:** PPP results at WUH2 over four 2 h arcs.

Scenario	Mean Used Satellites	PDOP	$t_{0.30}$ (min)	$t_{0.10}$ (min)	RMSE (m)
GPS	7.99	1.97	21.25	75.60	0.269
GPS + reference LEO	14.28	1.28	2.25	3.50	0.056
GPS + optimized LEO	15.16	1.27	2.00	3.00	0.057

## Data Availability

The LEO constellation data are openly accessible from internet. The codes for this study are available from Zixuan Rui upon reasonable request.
